# A Common Polymorphism in the *MTHFD1* Gene Is a Modulator of Risk of Congenital Heart Disease

**DOI:** 10.3390/jcdd9060166

**Published:** 2022-05-24

**Authors:** Nataša Karas Kuželički, Alenka Šmid, Maša Vidmar Golja, Tina Kek, Borut Geršak, Uroš Mazič, Irena Mlinarič-Raščan, Ksenija Geršak

**Affiliations:** 1 Department of Clinical Biochemistry, Faculty of Pharmacy, University of Ljubljana, Aškerčeva Cesta 7, 1000 Ljubljana, Slovenia; natasa.karas@ffa.uni-lj.si (N.K.K.); alenka.smid@ffa.uni-lj.si (A.Š.); masa1991@gmail.com (M.V.G.); irena.mlinaric@ffa.uni-lj.si (I.M.-R.); 2 Department of Obstetrics and Gynaecology, University Medical Centre Ljubljana, Zaloška 2, 1000 Ljubljana, Slovenia; tina.kek@gmail.com; 3 Faculty of Medicine, University of Ljubljana, Vrazov trg 2, 1000 Ljubljana, Slovenia; bgersak@icloud.com; 4 University Children’s Hospital, University Medical Centre Ljubljana, Bohoričeva 20, 1000 Ljubljana, Slovenia; uros.mazic@mf.uni-lj.si

**Keywords:** congenital heart defects, methylene-tetrahydrofolate dehydrogenase 1, folate supplementation, genetic risk factors

## Abstract

Several environmental and genetic factors may influence the risk of congenital heart defects (CHDs), which can have a substantial impact on pediatric morbidity and mortality. We investigated the association of polymorphisms in the genes of the folate and methionine pathways with CHDs using different strategies: a case–control, mother–child pair design, and a family-based association study. The polymorphism rs2236225 in the *MTHFD1* was confirmed as an important modulator of CHD risk in both, whereas polymorphisms in *MTRR*, *FPGS*, and *SLC19A1* were identified as risk factors in only one of the models. A strong synergistic effect on the development of CHDs was detected for *MTHFD1* polymorphism and a lack of maternal folate supplementation during early pregnancy. A common polymorphism in the *MTHFD1* is a genetic risk factor for the development of CHD, especially in the absence of folate supplementation in early pregnancy.

## 1. Introduction

Congenital heart defects (CHDs) occur in approximately nine per 1000 births, and they are thus among the more common congenital malformations [1]. Although CHDs can vary from relatively mild to very severe, they can have a significant impact on pediatric morbidity and mortality [2]. CHDs are a very heterogeneous group of diseases, and several systems for their classification have been proposed, including according to symptoms (e.g., cyanotic and a-cyanotic) [3,4], etiology (e.g., the National Birth Defect Prevention Study [NBDPS]) [2], and the ICD-10 World Health Organization classification [5].

Several environmental CHD risk factors have been identified [6], such as maternal folic acid deficiency, maternal diabetes, fever in first trimester, maternal chronic disease, advanced maternal age, and maternal drug exposure. However, the genetics of CHDs remain obscure, including for the numerous genes with weak contributions [7], and the sometimes conflicting effects in different studies.

**Figure 1:**
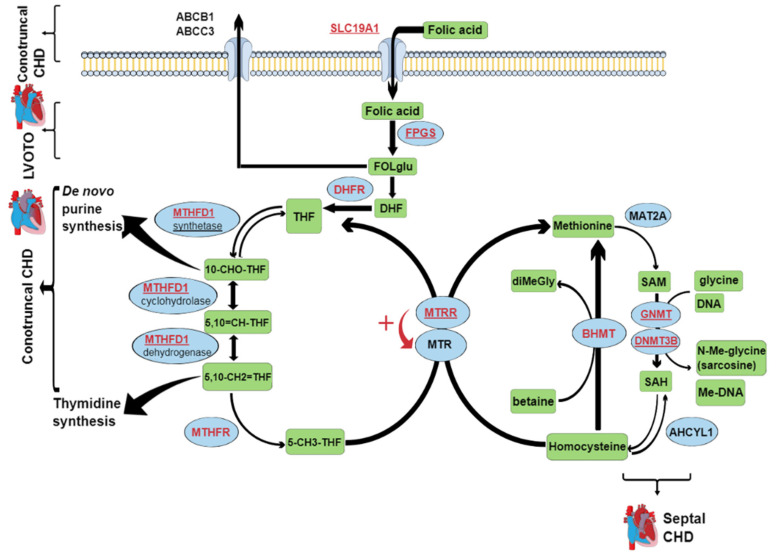
The folate metabolic pathways. Blue ellipses, enzymes; green rectangles, metabolites; written in red, names of genes selected for genotype analysis; underlined, genes that showed a significant association with certain types of CHD (contruncal, septal, and LVOTO). ABCB1, P-glycoprotein; ABCC3, multidrug resistant protein 3; *SLC19A1*; solute-carrier family 19; *FPGS*, folypolyglutamyl synthase; FOLglu, polyglutamylated folic acid; DHF, dihydrofolate; *DHFR*, dihydrofolate reductase; THF, tetrahydrofolate; *MTHFD1*, trifunctional methylenetetrahydrofolate dehydrogenase/cyclohydrolase/synthase; 10-CHO-THF, 10-formyl tetrahydrofolate; 5,10 = CH-THF, methenyl tetrahydrofolate; 5,10-CH2 = THF, methylene tetrahydrofolate; 5-CH3-THF, 5-methyl tetrahydrofolate; MTR, 5-methyl tetrahydrofolate-homocysteine methyltransferase; *MTRR*, 5-methyl tetrahydrofolate-homocysteine methyltransferase reductase; *MAT2A*, methionine adenosyltransferase II alpha; SAM, S-adenosylmethionine; SAH, S-adenosylhomocysteine; *GNMT*, glycine N-methyltransferase; *DNMT3B*, DNA (cytosine-5-)-methyltransferase 3 beta; *ACHYL1*, S-adenosylhomocysteine hydrolase-like 1; *BHMT*, betaine-homocysteine S-methyltransferase; diMeGly, dimethylglycine; Me, methyl; LVOTO, left ventricular outflow tract obstruction; CHD, congenital heart defect.

The role of folates and their metabolism in the development of neural tube defects (NTDs) is well established, although a firm connection between maternal folate supplementation during pregnancy and decreased risk of certain forms of CHDs was only recently confirmed [8]. The same association has been defined between food fortification with folic acid and reduction in the birth prevalence of specific CHDs [9]. An intact folate metabolism is crucial during embryo development, as it provides cells with enough one-carbon-activated folate cofactors for sufficient de novo purine and thymidine synthesis and for the production of the essential amino acid methionine and the elimination of the teratogenic homocysteine (Figure 1). In addition, the methionine cycle provides the main methyl donor S-adenosylmethionine, which is crucial to most remethylation reactions (Figure 1), including the DNA methylation that is an important regulator of gene expression during embryogenesis.

Polymorphisms in several genes involved in the folate and methionine cycles have been implicated as genetic risk factors for the development of CHDs, including rs1801131 and rs1801133 in the methylene-tetrahydrofolate reductase (*MTHFR*) gene [10], rs2236225 in the methylene-tetrahydrofolate dehydrogenase 1 (*MTHFD1*) gene [11], rs1801394 in the methyl-tetrahydrofolate-homocysteine methyltransferase reductase (*MTRR)* gene [12], rs1051266 in the solute carrier family 19 member 1 *(SLC19A1*) gene [13], duplications [14] and deletions [15] in the dihydrofolate reductase (*DHFR*) gene, and rs3733890 in the betaine-homocysteine S-methyltransferase (*BHMT*) gene [16]. However, the results from these studies have often been conflicting, as sometimes the same polymorphism has been positively or negatively correlated with, or even not at all associated with, CHDs. Furthermore, there are genes in the folate and methionine cycles and the connected methylation pathways that have never been studied in connection with CHDs, such as the genes for folypolyglutamyl synthase (*FPGS*), glycine N-methyltransferase (*GNMT*), and DNA (cytosine-5-)-methyltransferase 3 beta (*DNMT3B*). Thus, we undertook strategies to investigate the associations of these polymorphisms and genes with CHDs: a case–control, mother–child pair design, and a family-based association study. To our knowledge, this is the first study to investigate the influence on the development of CHDs of polymorphisms in *FPGS*, *GNMT*, and *DNMT3B*.

## 2. Materials and Methods

### 2.1. Study Cohort

The study cohort consisted of 199 pairs of children (cases) with CHD and their mothers and of 99 pairs of healthy children (controls) and their mothers. Since controls and cases were not paired, all multinomial logistic regression models were adjusted for demographic variables. In some CHD cases, the samples from the fathers were available, such that a total 44 family triads where the child was affected with CHD were collected. The DNA was collected from the children and their mothers and fathers using buccal swabbing. In addition, all of the mothers filled out a questionnaire about the potential demographic and environmental risk factors during their pregnancy with the index child. CHD cases were recruited sequentially. The children with CHD and their mothers were recruited at the Department of Cardiology, University Children`s Hospital, University Medical Centre Ljubljana (Slovenia), during routine check-ups. The buccal swabs from the fathers were obtained by post after they had consented to being involved in the study. The control samples were obtained from healthy newborns and their mothers at the Department of Obstetrics and Gynaecology, University Medical Centre Ljubljana, during their routine postnatal 3-day stays.

Enrolment time for the controls was two months and for CHD cases was 1.5 years. Since the study endpoint was the presence or absence of CHD, the follow-up time for CHD cases was not applicable. Controls were followed-up for a year after the inclusion in the study to assure the absence of any milder forms of CHD that might be detected later after birth.

Informed consent was obtained from all of the participants and/or their legal guardians. The study was approved by the National Medical Ethics Committee of the Republic of Slovenia (NMEC) (No. 57/02/13) and was performed in accordance with the relevant guidelines and regulations.

### 2.2. Questionnaire

The questionnaire that was completed by all of the mothers for both the case and control children consisted of two parts. The first part focused on exposure to known demographic and environmental risk factors, where the following data were collected: maternal age at conception, height, weight, smoking status, education, number of pregnancies, live births and miscarriages, family anamnesis of CHD and other congenital malformations, child gender, gestational diabetes, other chronic diseases of the mothers, drug and sauna use during pregnancy, fever during pregnancy, and folate and vitamin supplementation before conception and during pregnancy. The second part of the questionnaire was based on the Willett/Harvard food frequency questionnaire [17], and this was used to evaluate the maternal diet in the periconception period. The mothers were asked to recall their diet over the previous 4 weeks. They then reported on the similarity of their food intake during this previous 4 weeks to that in the periconception period. This used a scale ranging from 0 to 5, with 0 designating no recall or total discordance, and 5 denoting a high level of concordance. Based on this data, the monthly intake of folic acid and methionine in the periconception period was calculated for the mothers that reported levels 4 or 5 for diet concordance.

### 2.3. DNA Extraction and Genotyping

The DNA was extracted from buccal swabs using QIAamp DNA mini kits (Qiagen) or MasterPure complete DNA and RNA purification kits (Epicentre (Illumina) Madison, WI, USA), according to the manufacturer instructions.

The interactions between the genes of interest were evaluated by text and database mining using STRING 10.0 [18]. For each of the selected genes, at least one polymorphism was chosen for the genotype analysis, which showed a minor allele frequency ≥25% and the highest number of PubMed connections to CHD and/or etiologically related conditions (e.g., NTD and orofacial cleft). Ten common polymorphisms in nine genes involved in the folate and methionine cycles were analyzed using the TaqMan (Applied Biosystems, Foster City, CA, USA) or LightSNiP (TIB MOLBIOL, Berlin, Germany) probes, according to the manufacturer instructions. The following polymorphisms were genotyped using TaqMan probes: rs1544105 (*FPGS*) (assay number C_8342611_10), rs1677693 (DHFR) (assay number C_3103231_10), rs1801133 and rs1801131 (*MTHFR*) (assay numbers C_1202883_20 and C_850486_20), rs1801394 (*MTRR*) (assay number C_3068176_10), rs2236225 (*MTHFD1*) (assay number C_1376137_10), rs3733890 (*BHMT*) (assay number C_11646606_20), rs10948059 (*GNMT*) (assay number C_11425842_10), and rs2424913 (*DNMT3B*) (assay number C_25620192_20). Genotyping of rs1051266 (*SLC19A1*) was carried out by LightSNiP.

### 2.4. Statistical Analysis

#### 2.4.1. Analysis of Case and Control Mother–Child Pairs

The sample size calculation was based on the reported frequencies of the polymorphisms investigated in Caucasian populations and the detection of a 15% difference between wild-type and variant genotypes at 80% power and α = 0.05.

For continuous variables, the normality of the distribution across four categories (control, septal CHD, left ventricular outflow tract obstruction [LVOTO] CHD, and conotruncal CHD) was checked using Shapiro–Wilk tests. For simple statistical analysis, one-way ANOVA was used for Gaussian continuous variables, Kruskal–Wallis tests were used for non-Gaussian continuous and rank/score variables, and Fisher`s exact tests were used for categorical variables.

For all genotypes (except MTHFR), the dominant, recessive, and additive genetic models were calculated, although only the one with the highest statistical significance was used here. For MTHFR, two relevant polymorphisms were investigated (c.677 C > T; c.1298 A > C), which were analyzed together as genotype combinations. Using this approach, all of the subjects were classified into six genotype combinations. For the statistical analysis, the subjects were segregated into two groups according to the total number of mutated alleles at both loci: the wild-type genotype at both loci (i.e., 677 CC/1298 AA) and all of the other genotype combinations with at least one mutated allele (i.e., 677CT/1298AA, 677CC/1298AC, 677CC/1298CC, 677CT/1298AC, and 677TT/1298AA).

Odds ratios (ORs), 95% confidence intervals, and adjusted *p* values were calculated in multinomial logistic regression models for the mothers and children separately. Separate logistic regression models for the mothers and children were constructed to avoid violation of the assumption of no interactions. Only the variables with unadjusted *p* values < 0.250 were included in the multinomial logistic regression models. These variables were also adjusted for co-variables.

All of the tests were two-tailed, with the level of significance set at α = 0.05 for multinomial logistic regression. For the one-way ANOVA, Kruskal–Wallis, and Fisher’s exact tests, Bonferroni corrections for multiple testing were used, and *p* < 0.001 was considered significant.

All of the statistical analyses were performed using IBM SPSS Statistics 25.

#### 2.4.2. Likelihood Ratio Test Analysis

To test for association between CHD and the genetic markers studied, likelihood ratio tests (LRTs) were used, as developed by Fan et al. [19], which allows the use of triads, parent-child dyads, and singleton monads in a unified analysis. The associations were tested for each of the single nucleotide polymorphisms with CHD (all) or with subgroups of CHD (e.g., atrial septal defect, conotruncal, LVOTO, patent ductus arteriosus, and septal and ventricular septal defects) using three different models: dominant, recessive, and additive. All of the tests were performed using the statistical package R. The R codes were obtained from the Internet [20] and appropriately adjusted.

## 3. Results

### 3.1. Study Cohort Description

Of the 199 CHD patients recruited to the study, 113 (56.8%) were male, and 86 (43.2%) were female children; similarly, of the 199 children in the control group, 111 (55.8%) were male, and 88 (44.2%) were female. All of the mothers of both the cases and controls were included in the study, as well as 44 fathers of the CHD cases.

The full classification of the study cohort by CHD symptoms and etiology, and according to ICD-10 (WHO 2016), is given in Table 1. More than one of the malformations given in Table 1 was present in 48 (24%) of the cases.

### 3.2. Case–Control Study

The case–control study included the 199 CHD cases and their mothers as compared to the 199 control children and their mothers. The comparisons of the children and the mothers were carried out separately for genetic risk factors. First, all of the CHD cases were compared to the controls (i.e., irrespective of CHD type). Next, three of the most common NBDPS CHD classes were compared to the controls (i.e., septal CHD, conotruncal CHD, and LVOTO CHD). Finally, the most common types of the NBDPS CHD classes were compared to the controls (i.e., ventricular septal defect [VSD], atrial septal defect [ASD], aortic stenosis [AS], and tetralogy of Fallot [TOF]).

#### 3.2.1. CHD versus Controls

Among the environmental risk factors, only positive family anamnesis of CHD and not taking folate supplements in the first trimester of pregnancy were associated with the increased risk of CHD after Bonferroni correction for multiple testing (α = 0.001) and after adjustment for confounding variables in the logistic regression model. None of the genetic risk factors reached the threshold of significance of α = 0.001. The complete data of these relatively simple statistical and logistic regression analyses are presented in Table S1, Supplementary Materials.

#### 3.2.2. Septal, Conotruncal, and LVOTO CHD versus Controls

Using simple statistical analysis and after Bonferroni correction for multiple testing (α = 0.001), the following environmental risk factors reached the threshold of significance: child gender, number of pregnancies and live births, family anamnesis of CHD, and methionine and folic acid intake per month. The complete data for this relatively simple statistical analysis are presented in Table S2.

Multinomial logistic regression analysis was then performed for environmental risk factors. Only the variables with *p* < 0.250 were included in the model. In short, the risk for septal CHD was increased by the following: being female, maternal smoking, higher parity, positive family anamnesis of CHD, maternal chronic disease, and no intake of folates in early pregnancy. The risk factors for LVOTO were as follows: being male, higher parity, positive family anamnesis of CHD, and no intake of folates in early pregnancy. For conotruncal CHD, the only environmental risk factors identified were as follows: maternal smoking, higher parity, and positive family anamnesis of CHD (Table 2).

Multinomial logistic regression analysis was also performed for genetic risk factors that reached *p* < 0.250 in the simple statistical tests. Here, the maternal and child genotypes were analyzed in separate multinomial logistic regression models, which were adjusted for the environmental risk factors in Table 2. In multiple logistic regression analysis, no maternal or fetal genetic risk factors for septal and LVOTO CHDs were identified. On the other hand, the presence of genotypes *MTHFD1 rs2236225* GG or *MTRR rs1801394* AA in a child increased the risk of conotruncal CHD (Table 3).

### 3.3. Family Triads Study

Next, a different study design was used to confirm the data from the case–control study. In 44 family triads (i.e., CHD-affected child and his/her parents), the over-transmission of alleles from the unaffected parent to the affected child was investigated using LRTs. These LRTs were performed for CHD irrespective of the class, and separately for septal, LVOTO, and conotruncal CHD, using different genetic models (i.e., dominant, recessive, and additive). The statistically significant findings of these LRT analyses are given in Table 4.

The *MTHFD1* rs2236225 GG genotype was over-transmitted in the children with CHD for the analysis of both total CHD and conotruncal CHD, which confirmed the results of the case–control study. Conversely, the association of the *MTRR* rs1801394 AA genotype that was identified in the case–control study was not replicated. Additionally, an association was detected for *FPGS* rs1544105 T allele and *SLC19A1* rs1051266 A allele with LVOTO and conotruncal defects, respectively. This association was not seen in the case–control study.

### 3.4. Gene × Environment Interactions

**Figure 2:**
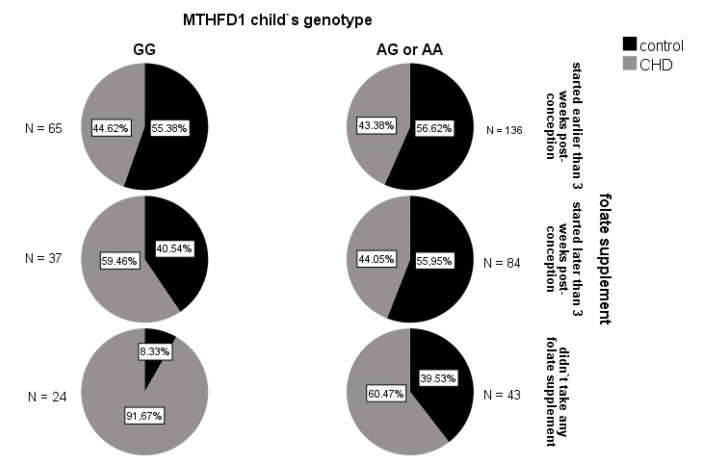
Incidence of CHD in the subgroups of the children, according to *MTHFD1* genotype and maternal folate supplementation in early pregnancy. The highest incidence of CHD (91.7%) was seen for the *MTHFD1* rs2236225 GG children whose mothers did not take any folate supplements. In contrast, the lowest incidences (~44%) were seen for the children of mothers who started folate intake early, irrespective of the *MTHFD1* genotype, and in the *MTHFD1* AG/AA children of mothers who started folate intake later than 3 weeks post conception.

To investigate whether there were any synergistic influences of the *MTHFD1* genotype and folate intake in early pregnancy on the overall CHD risk, the interaction term was included in the basic logistic regression model (see Section 3.2.1). A statistically significant interaction was detected between the *MTHFD1* genotype of the child and folate intake during early pregnancy. Namely, the odds ratio of CHD occurrence in *MTHFD1* GG versus AG/AA children of the group where the mothers did not use folates was 6.8-fold (95% CI 1.3–36.7; *p* = 0.025) compared to when the mothers started using folate supplement earlier than 3 weeks post-conception (Figure 2).

## 4. Discussion

The aim of the present study was to identify possible genetic risk factors for CHD, with a focus on folate and methionine metabolism. In addition to the genetic polymorphisms investigated, known environmental risk factors were also taken into account in the data analysis, to avoid bias. All of the environmental CHD risk factors that were identified in the present study (i.e., child gender, maternal smoking, higher parity, positive family anamnesis of CHD, maternal chronic disease, and a lack of folate supplementation during early pregnancy) had already been detected in previous studies [8,9,21,22,23]. We investigated the association of 10 common polymorphisms in nine genes that code for the enzymes and transporters in the folate-methionine metabolic pathways with CHD and its subtypes. Although the association of six polymorphisms (i.e., *MTHFD1* rs2236225, *MTRR* rs1801394, *SLC19A1* rs1051266, *GNMT* rs10948059, *DNMT3B* rs2424913, and *FPGS* rs1544105) with CHD was detected, the only polymorphism that was consistently associated with CHD (particularly conotruncal CHD) after correction for multiple testing and adjustment for environmental factors was *MTHFD1* rs2236225, as seen for both the case–control and family triads study designs. Thus, we can be confident that this finding represents a true biological association, and that it did not occur by chance. Table 5 gives the comparison of the data from the present study with data from previous studies of the selected polymorphisms.

As evident from the data given in Table 5, the results from the studies that have investigated the association of *MTHFD1* rs2236225 with CHD are ambiguous. The majority of the studies found no correlations between this polymorphism and CHD occurrence [15,24,25,26], and only one study found that AA is a risk genotype for CHD [11]. In contrast, the present study shows an increased risk of CHD in GG children, as well as an over-transmission of the G allele from unaffected parents to affected children. This is not surprising, as all of the above-mentioned studies included relatively small numbers of individuals. At the moment, the number of studies investigating associations between rs2236225 and CHD is too low to objectively evaluate the influence of rs2236225 on CHD development. In contrast, there are more studies that have investigated the influence of *MTHFD1* rs2236225 on NTDs, which are congenital malformations with similar etiopathogenesis. A recent meta-analysis including 2132 children with NTD and 4082 healthy controls, and this showed no association of rs2236225 with NTD, while in mothers of the NTD cases (n = 1402) and the control children (n = 3136), the AA genotype increased the NTD risk in their offspring. Interestingly, in the same meta-analysis, the GG genotype in fathers increased the risk of NTD in their children (993 case, 2879 control fathers) [27]. Of note, the rs2236225 G allele was also seen to increase the risk of type II diabetes [28] and lung cancer [29] and was associated with higher hyperactivity and impulsivity scores in children with attention-deficit disorder [30].

Another reason for the ambiguous data across these *MTHFD1* rs2236225 studies, apart from the small sample sizes, might be that the metabolic commitment of *MTHFD1* is strongly modulated by the cellular levels of folate, which can greatly vary among populations and individuals. *MTHFD1* is a trifunctional enzyme, with dehydrogenase, cyclohydrolase, and synthetase activities (Figure 1). *MTHFD1* is involved in two key metabolic pathways: thymidine synthesis, which takes place in the cell nucleus, and homocysteine re-methylation, which takes place in the cytosol [31]. In mammalian cells, nuclear translocation of the enzymes of thymidylate synthesis (including *MTHFD1*) is enabled through their linking to the small ubiquitin-like modifier SUMO [31]. Thus, thymidylate synthesis and re-methylation pathways compete for a limiting pool of methylenetetrahydrofolate cofactors, as does the *MTHFD1* enzyme [31,32]. In folate deficiency, *MTHFD1* is preferentially located in the nucleus. In this way, thymidylate synthesis is ensured, but at the expense of homocysteine re-methylation [31,32]. However, this effect is less pronounced for *MTHFD1* deficiency [32]. The total absence of *MTHFD1* activity has severe consequences, as *MTHFD1* knock-out mouse embryos (−/−) die at the early stage of gestation, while *MTHFD1* +/− females have an increased risk of malformed offspring [33]. However, such severe defects of *MTHFD1* are extremely rare in general human populations, while polymorphisms that can cause moderate decreases in *MTHFD1* activity are relatively common. One of the most investigated polymorphisms of *MTHFD1* is rs2236225, which leads to enzyme thermolability and consequently decreased enzyme activity. Thermolability can be prevented by addition of magnesium adenosine triphosphate or folate [11]. This can explain the interaction between the *MTHFD1* rs2236225 genotype and folate supplementation in early pregnancy that was detected in the present study (Figure 2). As in the present study, G alleles corresponding to higher *MTHFD1* activity increased CHD risk, and this might also be explained by an interaction mechanism. According to the data obtained for mouse models by two independent research groups [32,34], the test mice with moderately decreased *MTHFD1* activity had higher methylation potential in their cells, which indicated a higher flux through the homocysteine re-methylation pathway compared to the wild-type mice. This indicates that, in individuals with higher *MTHFD1* activity (i.e., the GG genotype), the thymidylate rescue mechanism is more effective, which results in higher thymidylate synthesis rates, but lower homocysteine re-methylation rates, and consequently higher intracellular levels of the teratogenic homocysteine. In the mouse models previously mentioned [32,34], the test mice with defective *MTHFD1* also had lower uracil mis-incorporation rates into DNA compared to the wild-type mice, which was probably due to the higher dUMP-to-dTMP methylation rate. In analogy, the rs2236225 GG individuals might have higher uracil mis-incorporation rates, which will lead to higher mutation rates and DNA damage, thus increasing the risk of congenital malformations (e.g., CHD).

The discrepancies in identified correlations between ours and other similar studies could be related to the differences among the studied populations. It is known that folate intake can influence the pathogenicity of mutations in genes coding for enzymes and transporters in the folate pathway. Since different populations have different diets and consequently differing folate intake and levels, the pathogenicity of those mutations can be expressed differently in different populations. The second populational influence could be the fact that *MAF* of rs2236225 varies among populations. For example, rs2236225 *MAF* is much higher in European and South Asian populations compared to those of East Asia and Africa.

## 5. Conclusions

We have shown that the common rs2236225 polymorphism in the *MTHFD1* gene is an important modulator of CHD risk, especially under conditions of folate deficiency. The results of similar studies have been ambiguous, probably due to the small sample sizes and complex nature of the *MTHFD1* metabolic pathways and its compartmentalization between the cell nucleus and cytosol under different folate levels. The limitation of the present study is again the relatively small sample size. However, *MTHFD1* rs2236225 was here identified as a CHD risk factor in both of the different study designs (i.e., case–control and family triads), which might at least in part compensate for this limitation.

## Figures and Tables

**Table 1:** **Classification of the study cohort by CHD symptoms and etiology, and according to ICD-10 (WHO 2016).** ACY, acyanotic; CY, cyanotic; NBDPS, National Birth Defect Prevention Study (CDC coordinated USA nationwide study); LVOTO, left ventricular outflow tract obstruction; PDA, patent ductus arteriosus; RVOTO, right ventricular outflow tract obstruction; AVSD, atrioventricular septal defect; Q20, congenital malformations of the cardiac chambers and connections; Q21, congenital malformations of the cardiac septa; Q22, congenital malformations of the pulmonary and tricuspid valves; Q23, congenital malformations of the aortic and mitral valves; Q24, other congenital malformations of the heart; Q25, congenital malformations of the great arteries.

CHD Type	Cases [n (%)]	Classification		
		By Symptoms	By Aetiology (NBDPS Level 3)	ICD-10
Ventricular septal defect	80 (40)	ACY	Septal	Q21.0
Atrial septal defect	60 (30)	ACY	Septal	Q21.1
Aortic stenosis	21 (11)	ACY	LVOTO	Q25.3
Patent ductus arteriosus	16 (8)	ACY	PDA	Q25.0
Coarctation of aorta	15 (8)	ACY	LVOTO	Q25.1
Tetralogy of Fallot	13 (7)	CY	Conotruncal	Q21.3
Bicuspid aortic valve	10 (5)		LVOTO	Q23.1
Pulmonary valve stenosis	8 (4)	ACY	RVOTO	Q22.1
Transposition of great vessels	7 (4)	CY	Conotruncal	Q20.3
Atrioventricular septal defect	5 (3)		AVSD	Q21.2
Double outlet right ventricle	5 (3)	CY	Conotruncal	Q20.1
Hypoplastic left heart syndrome	3 (2)		LVOTO	Q23.4
Pulmonary valve atresia	3 (2)	CY	RVOTO	Q22.0
Persistent truncus arteriosus	3 (2)	CY	Conotruncal	Q20.0
Mitral valve prolapse	2 (1)		LVOTO	Q23.8
Aortic regurgitation	1 (0.5)		LVOTO	Q23.1
Mitral (valve) stenosis	1 (0.5)	ACY	LVOTO	Q23.2
Tricuspid atresia	1 (0.5)	CY	RVOTO	Q22.4
Atrial septal aneurism	1 (0.5)			
Major aotropulmonary collateral artery	1 (0.5)			
Single ventricle	1 (0.5)		Complex	Q20.4
Overriding aorta	1 (0.5)		Conotruncal	Q25.4
Right atrial isomerism	1 (0.5)		Heterotaxy	Q20.6
Pulmonary artery stenosis	1 (0.5)		RVOTO	Q25.6
Mitral valve insufficiency	1 (0.5)		LVOTO	Q23.3
Mitral valve cleft	1 (0.5)		LVOTO	Q23.8

**Table 2:** Multinomial logistic regression models of selected environmental risk factors in the control and congenital heart defect (CHD) sub-groups (septal, left ventricular outflow tract obstruction [LVOTO], and conotruncal). OR, odds ratio; CI, confidence interval.

Variable	Control vs. Septal		Control vs. LVOTO		Control vs. Conotruncal	
	OR (95% CI) ^‡^	*p* _adj_^‡^	OR (95% CI) ^‡^	*p* _adj_^‡^	OR (95% CI) ^‡^	*p* _adj_^‡^
Child gender						
Male ^†^ vs. female	2.1 (1.1–4.1)	0.035	0.2 (0.1–0.7)	0.011	0.4 (0.1–1.2)	0.094
Maternal smoking status						
Non-smoker ^†^ vs. smoker	7.3 (2.1–24.9)	0.002	2.9 (0.7–11.8)	0.139	7.7 (1.8–31.9)	0.005
Non-smoker ^†^ vs. ex-smoker	2.7 (1.1–6.5)	0.029	0.7 (0.2–2.1)	0.523	1.4 (0.4–5.0)	0.561
Maternal education						
MSc, PhD ^†^ vs. elementary school	6.1 (0.5–72.3)	0.154	4.1 (0.1–127)	0.415	1.4 (0.04–45.1)	0.865
MSc, PhD ^†^ vs. vocational school	2.4 (0.4–13.1)	0.311	3.7 (0.3–47.9)	0.312	3.2 (0.3–30.7)	0.309
MSc, PhD ^†^ vs. high school	2.8 (0.3–23.4)	0.345	2.8 (0.1–75.6)	0.549	NA	
MSc, PhD ^†^ vs. college	4.6 (0.8–26.7)	0.090	3.9 (0.2–65.9)	0.339	0.7 (0.04–13.9)	0.812
MSc, PhD ^†^ vs. university	1.6 (0.3–8.6)	0.560	2.8 (0.2–35.8)	0.418	0.7 (0.06–7.2)	0.737
No. of live births	1.8 (1.2–2.6)	0.004	2.1 (1.4–3.3)	0.001	1.9 (1.1–3.1)	0.017
Family anamnesis of CHD						
Negative ^†^ vs. Positive	9.3 (2.3–37.0)	0.002	18.9 (3.8–90.9)	0.0003	11.1 (2.1–58.8)	0.005
Maternal chronic disease						
No ^†^ vs. Yes	2.8 (1.0–7.4)	0.043	3.6 (0.9–13.9)	0.062	0.9 (0.2–5.5)	0.921
Other drugs in pregnancy						
No ^†^ vs. Yes	2.0 (1.0–4.1)	0.055	3.6 (0.9–13.9)	0.062	0.9 (0.2–5.5)	0.921
Folate supplement initiation						
No folate suppl. ^†^ vs. before 3 weeks post-conception	0.3 (0.1–0.7)	0.010	0.2 (0.05–0.7)	0.011	0.6 (0.2–2.1)	0.394
No folate suppl. ^†^ vs. after 3 weeks post-conception	0.4 (0.1–1.1)	0.067	0.5 (0.1–2.0)	0.350	0.4 (0.1–2.0)	0.286
Folic acid intake per month (mg)	1.0 (1.0–1.0)	0.069	1.0 (1.0–1.0)	0.417	1.0 (1.0–1.0)	0.071

^†^ Reference category. ^‡^ Odds ratio (OR), 95% confidence interval (CI), and adjusted *p* values were calculated in multinomial logistic regression models for mothers and children separately. Only variables with unadjusted *p* values < 0.250 were included in the multinomial logistic regression models and adjusted for co-variables. Variables with high levels of correlation were not included in the same model. NA: not applicable (the variable was not tested in the specific model).

**Table 3:** Multinomial logistic regression models of maternal and children`s selected genetic risk factors in control and CHD sub-groups (septal, left ventricular outflow tract obstruction [LVOTO], and conotruncal). LVOTO, left ventricular outflow tract obstruction; OR, odds ratio; CI, confidence interval; *SLC19A1*, solute-carrier family 19; *FPGS*, folypolyglutamyl synthase; *GNMT*, glycine N-methyltransferase; *DNMT3B*, DNA (cytosine-5-)-methyltransferase 3 beta; *MTHFD1*, trifunctional methylenetetrahydrofolate dehydrogenase/cyclohydrolase/synthase; *MTRR*, 5-methyl tetrahydrofolate-homocysteine methyltransferase reductase.

Variable	Control vs. Septal		Control vs. LVOTO		Control vs. Conotruncal	
	OR (95% CI)	*p* _adj_^‡^	OR (95% CI)	*p* _adj_^‡^	OR (95% CI)	*p* _adj_^‡^
Child genotype *SLC19A1* rs1051266 GG ^†^ vs. AG or AA	0.6 (0.3–1.3)	0.178	0.6 (0.2–1.7)	0.358	1.7 (0.5–6.0)	0.389
Maternal genotype *FPGS* rs1544105 CC or CT ^†^ vs. TT	0.9 (0.4–2.0)	0.775	1.9 (0.7–5.6)	0.221	0.3 (0.05–1.5)	0.139
Maternal genotype *GNMT* rs10948059 CC or CT ^†^ vs. TT	1.5 (0.7–3.2)	0.316	0.8 (0.3–2.7)	0.745	1.6 (0.5–4.9)	0.402
Maternal genotype *DNMT3B* rs2424913 CC ^†^ vs. CT or TT	0.5 (0.3–1.1)	0.090	1.3 (0.4–3.8)	0.649	1.3 (0.4–4.0)	0.686
Child genotype *MTHFD1* rs2236225 GG ^†^ vs. AG or AA	0.8 (0.4–1.7)	0.543	0.9 (0.3–2.6)	0.895	0.2 (0.08–0.7)	0.007
Child genotype *MTHFR* 677 CC/1298 AA ^†^ vs. at least one mutated allele	0.8 (0.3–2.5)	0.761	0.7 (0.2–3.0)	0.647	0.4 (0.1–1.5)	0.190
Maternal genotype *MTRR* rs1801394 AA or AG ^†^ vs. GG	1.1 (0.5–2.2)	0.882	1.3 (0.5–3.4)	0.562	0.5 (0.2–1.6)	0.231
Child genotype *MTRR* rs1801394 AA ^†^ vs. AG or GG	0.9 (0.3–2.4)	0.838	0.7 (0.2–2.8)	0.660	0.2 (0.08–0.7)	0.013
Number of mutated alleles in mother-child pairs	1.0 (0.9–1.1)	0.695	1.0 (0.9–1.2)	0.941	0.9 (0.7–1.0)	0.095

^†^ Reference category. ^‡^ All genetic risk factors were adjusted for environmental risk factors listed in Table 2. Odds ratio (OR), 95% confidence interval, and adjusted *p* values were calculated in multinomial logistic regression models for mothers and children separately. Only variables with unadjusted *p* values < 0.250 were included in the multinomial logistic regression models and adjusted for co-variables.

**Table 4:** **Likelihood ratio test best hits in the family triads.** CHDs, congenital heart defects; LVOTO, left ventricular outflow tract obstruction; *FPGS*, folypolyglutamyl synthase; *SLC19A1*, solute carrier family 19 member 1; *MTHFD1*, trifunctional methylenetetrahydrofolate dehydrogenase/cyclohydrolase/synthase.

CHD Type	Gene	Single Nucleotide Polymorphism	Model	Number of Triads	Likelihood Ratio	*p* (α = 0.05)
CHD total	*MTHFD1*	rs2236225	Recessive	44	5.429	0.020
LVOTO	*FPGS*	rs1544105	Dominant	7	4.861	0.027
Conotruncal	*SLC19A1*	rs1051266	Dominant	4	4.052	0.044
	*MTHFD1*	rs2236225	Recessive	4	5.895	0.015

**Table 5:** Studies that have investigated the involvement of *MTHFD1* rs2236225, *MTRR* rs1801394, *SLC19A1* rs1051266, *GNMT* rs10948059, *DNMT3B* rs2424913, and *FPGS* rs1544105 in CHD development. *GNMT*, *DNMT3B*, and *FPGS* have not been studied in association with CHDs. *MTHFD1*, trifunctional methylenetetrahydrofolate dehydrogenase/cyclohydrolase/synthase; *MTRR*, methyl-tetrahydrofolate-homocysteine methyltransferase reductase; *GNMT*, glycine N-methyltransferase; *DNMT3B*, DNA (cytosine-5-)-methyltransferase 3 beta; *FPGS*, folypolyglutamyl synthase; SLC19A1, solute carrier family 19 member 1; CHDs, congenital heart defects.

Study	Study Design	Population	Number Cases/Controls	Gene	Single Nucleotide Polymorphism	CHD Risk Genotype or Allele
Christensen KE et al., 2008 [11]	Mother-child pair, case-control	N. European	Children: 158/110Mothers: 199/105	*MTHFD1*	rs2236225	AA (increased risk)
Zeng W et al., 2011 [35]	Case-control	Chinese Han	599/672	*MTRR*	rs1801394	GG (increased risk)
Cai B et al., 2014 [12]	Meta-analysis	Mixed	914/964441 families	*MTRR*	rs1801394	G allele (increased risk)
Pei L et al., 2006 [13]	Case-control, family based	Chinese	Families: 67/100	*SLC19A1*	rs1051266	G allele (increased risk)
Gong D et al., 2012. [24]	Case-control	Chinese Han	244/136	*MTHFD1*	rs2236225	No association
				*SLC19A1*	rs1051266	A allele (increased risk)
Christensen KE et al., 2013 [36]	Mother-child pair, case-control	N. European	Children: 156/69	*MTRR*	rs1801394	G allele (decreased risk)
			Mothers: 181/65	*SLC19A1*	rs1051266	No association
Wang B et al., 2013 [15]	Case-control	Chinese	160/188	*MTHFD1*	rs2236225	No association
				*MTRR*	rs1801394	No association
				*SLC19A1*	rs1051266	No association
Mitchell LE et al., 2010 [16]	Family based	Mixed	386 case-family triads	*MTRR*	rs1801394	No association
Goldmuntz E et al., 2008 [37]	Family based	Mixed	727 case-family triads	*MTRR*	rs1801394	No association
Huang J et al., 2014 [25]	Case-control	Chinese	173/2017	*MTHFD1*	rs2236225	No association (GG more prevalent in cases than controls)
Shaw GM et al., 2009 [26]	Case-control	Mixed	214/359	*MTHFD1*	rs2236225	No association
Guo KN et al., 2017 [38]	Parents of cases and controls	Chinese Han	99/114	*MTRR*	rs1801394	G allele (increased risk)
Yu D et al., 2014 [39]	Meta-analysis Asian	Caucasian	3.592/3.638	*MTRR*	rs1801394	G allele (increased risk)
Elizabeth KE et al., 2017 [40]	Mother-child pair, case-control	Indian	Pairs: 32/32	*MTRR*	rs1801394	G allele (increased risk)
Hassan FM et al., 2017 [41]	Case-control	Egyptian	100/100	*MTRR*	rs1801394	G allele (increased risk)
Present study	Case-control and family based	Caucasian	Case-control: 199/199Family triads: 44	*MTHFD1*	rs2236225	GG (increased risk)
				*MTRR*	rs1801394	AA (increased risk)
				*SLC19A1*	rs1051266	A allele (increased risk)
				*GNMT*	rs10948059	TT (increased risk)
				*DNMT3B*	rs2424913	CC (increased risk)
				*FPGS*	rs1544105	TT (increased risk)

## Data Availability

The data presented in this study are available from the corresponding author on request. The data are not publicly available due to privacy of the study participants.

## Supplementary Materials

The following supporting information can be downloaded at: https://www.mdpi.com/article/10.3390/jcdd9060166/s1: Table S1: Differences for all of the variables tested between the control and CHD groups using simple statistical tests and logistic regression models; Table S2: Differences in all tested variables between control and CHD etiologic sub-groups, calculated using simple statistical tests; Table S3: Statistically significant and marginally significant differences in genotypes in four most common septal (VSD & ASD), LVOTO (AS) and conotruncal (TOF) defects.
