# Supplementary material for: A Common Polymorphism in the MTHFD1 Gene Is a Modulator of Risk of Congenital Heart Disease
Source: J Cardiovasc Dev Dis. 2022 May 24;9(6):166. doi: 10.3390/jcdd9060166 (PMC9224796; doi:10.3390/jcdd9060166)
Supplement: Supplementary file 1 [file jcdd-09-00166-s001.zip › jcdd-1703713-supplementary.pdf]

## Supporting Information

**A common polymorphism in the methylene-tetrahydrofolate dehydrogenase 1 (*MTHFD1*) gene is a modulator of risk of congenital heart disease**

Nataša Karas Kuželički[1], Alenka Šmid[1], Maša Vidmar Golja[1,2], Tina Kek[2,3], Borut Geršak[3], Uroš Mazič[4], Irena Mlinarič-Raščan[1], Ksenija Geršak[2,3]

[1] Department of Clinical Biochemistry, Faculty of Pharmacy, University of Ljubljana, Aškerčeva 7, Ljubljana, Slovenia

[2] Department of Obstetrics and Gynaecology, University Medical Centre Ljubljana, Zaloska 2, Ljubljana, Slovenia

[3] Faculty of Medicine, University of Ljubljana, Vrazov trg 2, Ljubljana, Slovenia

[4] University Children`s Hospital, University Medical Centre Ljubljana, Bohoričeva 20, Ljubljana, Slovenia

**Supplementary Table S1.** Differences for all of the variables tested between the control and CHD groups using simple statistical tests and logistic regression models.

| Variable | Total (n) | Mean ±SD[a] Median (minimum, maximum)[b] or Frequency [n(%)][c] | | p unadjusted (α = 0.001) | Mother[d] (α = 0.05) | | Child[d] (α = 0.05) | |
|---|---|---|---|---|---|---|---|---|
| | | Control | CHD | | OR (95% CI) | p adjusted | OR (95% CI) | p adjusted |
| **Maternal height (cm)** | 394 | 167 ±6 | 167 ±6 | 0.842 | -- | -- | -- | -- |
| **Maternal weight at conception (kg)** | 398 | 62 (40-116) | 63 (43-132) | 0.269 | -- | -- | -- | -- |
| **Maternal age at conception (years)** | 391 | 31 ±5 | 30 ±5 | 0.076 | -- | -- | -- | -- |
| **Child gender** | 398 | | | 0.920 | | | | |
| Male | | 113 (56.8%) | 111 (55.8%) | | -- | -- | -- | -- |
| Female | | 86 (43.2%) | 88 (44.2%) | | | | | |
| **Maternal smoking status** | 398 | | | 0.020 | | | | |
| Non-smoker | | 112 (56.3%) | 109 (54.8%) | | | | | |
| Smoker | | 23 (11.6%) | 42 (21.1%) | | 3.3 (1.5-7.3) | 0.004 | 3.3 (1.5-7.3) | 0.004 |
| Ex-smoker | | 64 (32.2%) | 48 (24.1%) | | 0.8 (0.4-1.4) | 0.359 | 0.8 (0.4-1.4) | 0.359 |
| ***Maternal ex-smokers: time without smoking (years)*** | 106 | 2 (0.4-21) | 4 (0.1-23) | 0.003 (α = 0.05) | -- | -- | -- | -- |
| **Smoking in pregnancy** | 392 | | | | | | | |
| No | | 176 (88.9%) | 170 (87.6%) | 0.755 | -- | -- | -- | -- |
| Yes | | 22 (11.1%) | 24 (12.4%) | | | | | |
| **Maternal education** | 398 | | | 0.001 | | | | |
| Elementary | | 3 (1.5%) | 13 (6.5%) | | | | | |
| Vocational | | 63 (31.7%) | 93 (46.7%) | | 0.6 (0.1-3.0) | 0.539 | 0.6 (0.1-3.0) | 0.539 |
| High school | | 12 (6.0%) | 9 (4.5%) | | 0.5 (0.1-3.3) | 0.455 | 0.5 (0.1-3.3) | 0.455 |
| College | | 24 (12.1%) | 24 (12.1%) | | 0.6 (0.1-3.5) | 0.569 | 0.6 (0.1-3.5) | 0.569 |
| University | | 82 (41.2%) | 52 (26.1%) | | 0.4 (0.1-2.1) | 0.268 | 0.4 (0.1-2.1) | 0.268 |
| MSc or PhD | | 15 (7.5%) | 8 (4.0%) | | 0.3 (0.0-2.0) | 0.208 | 0.3 (0.0-2.0) | 0.208 |
| **No. of pregnancies** | 396 | 1 (1-6) | 2 (1-12) | <0.001 | -- | -- | -- | -- |
| **No. of live births** | 396 | 1 (1-4) | 2 (1-12) | <0.001 | -- | -- | -- | -- |
| **No. of miscarriages** | 396 | 0 (0-4) | 0 (0-3) | 0.014 | -- | -- | -- | -- |
| **Family anamnesis of CHD** | 398 | | | | | | | |

| | | | | | | | | |
|---|---|---|---|---|---|---|---|---|
| Negative | | 194 (97.5%) | 159 (79.9%) | $1.4 \times 10^{-8}$ | 11 (3-39) | $1.5 \times 10^{-4}$ | 11 (3-39) | $1.5 \times 10^{-4}$ |
| Positive | | 5 (2.5%) | 40 (20.1%) | | | | | |
| **Maternal diabetes at conception** | 398 | | | -- | | | | |
| No | | 199 (100%) | 199 (100%) | | -- | -- | -- | -- |
| Yes | | 0 (0%) | 0 (0%) | | | | | |
| **Gestational diabetes** | 398 | | | | | | | |
| No | | 171 (85.9%) | 175 (87.9%) | 0.656 | -- | -- | -- | -- |
| Yes | | 28 (14.1%) | 24 (12.1%) | | | | | |
| **Maternal chronic disease** | 398 | | | | | | | |
| No | | 184 (92.5%) | 161 (80.9%) | 0.001 | 3.1 (1.4-7.0) | 0.006 | 3.1 (1.4-7.0) | 0.006 |
| Yes | | 15 (7.5%) | 38 (19.1%) | | | | | |
| **Antiepileptics in pregnancy** | 398 | | | | | | | |
| No | | 198 (99.5%) | 198 (99.5%) | 1.000 | -- | -- | -- | -- |
| Yes | | 1 (0.5%) | 1 (0.5%) | | | | | |
| **Other drugs in pregnancy** | 398 | | | | | | | |
| No | | 107 (53.8%) | 87 (43.7%) | 0.057 | -- | -- | -- | -- |
| Yes | | 92 (46.2%) | 112 (56.3%) | | | | | |
| **Fever in pregnancy** | 398 | | | | | | | |
| No | | 186 (93.5%) | 190 (95.5%) | 0.511 | -- | -- | -- | -- |
| Yes | | 13 (6.5%) | 9 (4.5%) | | | | | |
| **Sauna in pregnancy** | 398 | | | | | | | |
| No | | 194 (97.5%) | 198 (99.5%) | 0.215 | -- | -- | -- | -- |
| Yes | | 5 (2.5%) | 1 (0.5%) | | | | | |
| **Folate supplement initiation** | 392 | | | | | | | |
| No folate suppl. | | 19 (9.8%) | 48 (24.2%) | $4.5 \times 10^{-4}$ | | | | |
| Before 3 wk p.c. | | 113 (58.2%) | 90 (45.5%) | | 0.3 (0.1-0.7) | 0.002 | 0.3 (0.1-0.7) | 0.002 |
| After 3 wk p.c. | | 62 (32%) | 60 (30.3%) | | 0.4 (0.2-0.8) | 0.019 | 0.4 (0.2-0.8) | 0.019 |
| ***Folate supplement in pregnancy: folate formulation*** | 284 | | | | | | | |
| Folic acid | | 134 (88.2%) | 119 (90.2%) | 0.704 | -- | -- | -- | -- |
| Metafolin | | 18 (11.8%) | 13 (9.8%) | ($\alpha = 0.05$) | | | | |
| **Other supplements in pregnancy**[e] | 398 | | | | | | | |
| No | | 158 (79.4%) | 143 (71.9%) | 0.102 | -- | -- | -- | -- |

| | | | | | | | | |
|---|---|---|---|---|---|---|---|---|
| Yes | | 42 (20.6%) | 56 (28.1%) | | | | | |
| **Methionine per month intake (g)** | 314 | 47 (18-847) | 36 (5-488) | <0.001 | -- | -- | -- | -- |
| **Folic acid per month intake (mg)** | 314 | 23 (8-511) | 19 (4-333) | <0.001 | 1.0 (1.0-1.0) | 0.078 | 1.0 (1.0-1.0) | 0.078 |
| **Maternal genotype SLC19A1** | 392 | | | | | | | |
| GG | | 70 (35.2%) | 67 (34.7%) | 1.000 | -- | -- | -- | -- |
| AG or AA | | 129 (64.8%) | 126 (65.3%) | | | | | |
| **Child genotype SLC19A1** | 392 | | | | | | | |
| GG | | 54 (27.1%) | 69 (35.8%) | 0.081 | -- | -- | 0.6 (0.4-1.1) | 0.105 |
| AG or AA | | 145 (72.9%) | 124 (64.2%) | | | | | |
| **Maternal genotype DHFR** | 394 | | | | | | | |
| CC | | 110 (55.3%) | 119 (61.0%) | 0.262 | -- | -- | -- | -- |
| CA or AA | | 89 (44.7%) | 76 (39.0%) | | | | | |
| **Child genotype DHFR** | 395 | | | | | | | |
| CC | | 111 (55.8%) | 112 (57.1%) | 0.839 | -- | -- | -- | -- |
| CA or AA | | 88 (44.2%) | 84 (42.9%) | | | | | |
| **Maternal genotype FPGS** | 395 | | | | | | | |
| CC | | 72 (36.2%) | 66 (33.7%) | 0.673 | -- | -- | -- | -- |
| CT or TT | | 127 (63.8%) | 130 (66.3%) | | | | | |
| **Child genotype FPGS** | 393 | | | | | | | |
| CC | | 70 (35.2%) | 65 (33.5%) | 0.751 | -- | -- | -- | -- |
| CT or TT | | 129 (64.8%) | 129 (66.5%) | | | | | |
| **Maternal genotype BHMT** | 394 | | | | | | | |
| GG or AG | | 184 (92.5%) | 178 (87.6%) | 0.715 | -- | -- | -- | -- |
| AA | | 15 (7.5%) | 17 (12.4%) | | | | | |
| **Child genotype BHMT** | 392 | | | | | | | |
| GG or AG | | 182 (91.9%) | 170 (92.5%) | 0.183 | -- | -- | 1.9 (0.8-4.5) | 0.145 |
| AA | | 16 (8.1%) | 24 (7.5%) | | | | | |
| **Maternal genotype GNMT** | 394 | | | | | | | |
| CC or CT | | 161(80.9%) | 141(72.3%) | 0.056 | 1.1 (0.6-2.0) | 0.827 | -- | -- |
| TT | | 38 (19.1%) | 54 (27.7%) | | | | | |
| **Child genotype GNMT** | 390 | | | | | | | |
| CC | | 65 (32.7%) | 51 (26.7%) | 0.223 | -- | -- | 0.9 (0.5-1.6) | 0.751 |
| CT or TT | | 134 (67.3%) | 140 (73.3%) | | | | | |

| | | | | | | | | |
|---|---|---|---|---|---|---|---|---|
| **Maternal genotype DNMT3B** | 394 | | | | | | | |
| CC or CT | | 149 (74.9%) | 156 (80.0%) | 0.231 | 0.7 (0.4-1.4) | 0.349 | -- | -- |
| TT | | 50 (25.1%) | 39 (20.0%) | | | | | |
| **Child genotype DNMT3B** | 394 | | | | | | | |
| CC or CT | | 156 (78.4%) | 163 (83.6%) | 0.201 | -- | -- | 0.7 (0.4-1.3) | 0.265 |
| TT | | 43 (21.6%) | 32 (16.4%) | | | | | |
| **Maternal genotype MTHFD1** | 393 | | | | | | | |
| GG | | 68 (34.3%) | 67 (34.4%) | 1.000 | -- | -- | -- | -- |
| AG or AA | | 130 (65.7%) | 128 (65.6%) | | | | | |
| **Child genotype MTHFD1** | 394 | | | | | | | |
| GG | | 54 (27.3%) | 74(37.8%) | 0.031 | -- | -- | 0.8 (0.4-1.3) | 0.350 |
| AG or AA | | 144 (72.7%) | 122 (62.2%) | | | | | |
| **Maternal genotype MTHFR** | 395 | | | | | | | |
| 677 CC/1298 AA | | 30 (15.2%) | 23 (11.7%) | 0.376 | -- | -- | -- | -- |
| At least one mutated allele | | 168 (84.8%) | 174 (88.3%) | | | | | |
| **Child genotype MTHFR** | 396 | | | | | | | |
| 677 CC/1298 AA | | 16 (8.1%) | 26 (13.1%) | 0.141 | -- | -- | 0.6 (0.3-1.4) | 0.237 |
| At least one mutated allele | | 182 (91.9%) | 172 (86.9%) | | | | | |
| **Maternal genotype MTRR** | 396 | | | | | | | |
| AA | | 32 (16.2%) | 38 (19.2%) | 0.510 | -- | -- | -- | -- |
| AG or GG | | 166 (83.8%) | 160 (80.8%) | | | | | |
| **Child genotype MTRR** | 395 | | | | | | | |
| AA | | 30 (15.2%) | 34 (17.3%) | 0.588 | -- | -- | -- | -- |
| AG or GG | | 168 (84.8%) | 163 (82.7%) | | | | | |
| **No. of mutated alleles in mother–child pairs[f]** | 387 | 16 (8-27) | 16 (7-27) | 0.847 | -- | -- | -- | -- |

Italic text, variables tested only in subgroups. Discrepancies between the total numbers of subjects for different variables are due to the missing questionnaire data or unsuccessful genotyping.

[a], Gaussian parametric variables: for simple statistical analysis (unadjusted p) independent samples t-tests were used.

[b], Non-Gaussian parametric and rank/ score variables: for simple statistical analysis (unadjusted p) Mann-Whitney U tests were used.

[c], Categorical variables: for simple statistical analysis (unadjusted p) Fisher`s exact tests were used. In the logistic regression models, the first listed category is the reference category.

[d], Odds ratio (OR), 95% confidence interval, and adjusted p values were calculated using logistic regression models for mothers and children separately. Only variables with unadjusted p values <0.250 were included in the logistic regression models, and adjusted for co-variables. Some variables were not included in the logistic regression analysis due to the high level of correlation with other variables in the logistic regression model.

[e], Multivitamins, minerals (Mg, Ca), omega 3.

[f], Minimum, 0; maximum, 40.

--, not applicable. The variable was not included in the logistic regression models.

**Supplementary Table S2.** Differences in all tested variables between control and CHD etiologic sub-groups, calculated using simple statistical tests.

| **Variable** | **Total (n)** | **Mean ± SD[a] Median (minimum, maximum)[b] or Frequency (N(%))[c]** | | | | **$p_{unadjusted}$ α = 0.001** |
|---|---|---|---|---|---|---|
| | | **Control** | **CHD Septal** | **CHD LVOTO** | **CHD Conotruncal** | |
| **Maternal height (cm)** | 354 | 167 ± 6 | 167 ± 6 | 167 ± 7 | 167 ± 5 | 0.971 |
| **Maternal weight at conception (kg)** | 358 | 62 (40-116) | 61 (45-132) | 64 (48-116) | 65 (45-101) | 0.403 |
| **Maternal age at conception (years)** | 351 | 31 ± 5 | 30 ± 4 | 30 ± 6 | 30 ± 6 | 0.570 |
| **Child gender** | 358 | | | | | |
| Male | | 113 (56.8%) | 38 (41.3%) | 33 (84.6%) | 22 (78.6%) | $4.0 \times 10^{-6}$ |
| Female | | 86 (43.2%) | 54 (58.7%) | 6 (15.4%) | 6 (21.4%) | |
| **Maternal smoking status** | 358 | | | | | |
| Non-smoker | | 112 (56.3%) | 61 (66.3%) | 16 (41.0%) | 12 (42.9%) | 0.001 |
| Smoker | | 23 (11.6%) | 15 (16.3%) | 8 (20.5%) | 11 (39.3%) | |
| Ex-smoker | | 64 (32.2%) | 16 (17.4%) | 15 (38.5%) | 5 (17.9%) | |
| **Smoking in pregnancy** | 353 | | | | | |
| No | | 176 (88.9%) | 79 (87.8%) | 33 (89.2%) | 25 (89.3%) | 0.992 |
| Yes | | 22 (11.1%) | 11 (12.2%) | 4 (10.8%) | 3 (10.7%) | |
| **Maternal education** | 358 | | | | | |
| Elementary | | 3 (1.5%) | 7 (7.6%) | 4 (10.3%) | 1 (3.6%) | 0.001 |
| Vocational | | 63 (31.7%) | 36 (39.1%) | 20 (51.3%) | 21 (75.0%) | |
| High school | | 12 (6.0%) | 5 (5.4%) | 2 (5.1%) | 0 (0.0%) | |
| College | | 24 (12.1%) | 15 (16.3%) | 4 (10.3%) | 1 (3.6%) | |
| University | | 82 (41.2%) | 25 (27.2%) | 8 (20.5%) | 4 (14.3%) | |
| MSc or PhD | | 15 (7.5%) | 4 (4.3%) | 1 (2.6%) | 1 (3.6%) | |
| **No. of pregnancies** | 356 | 1 (1-6) | 2 (1-8) | 2 (1-12) | 3 (1-4) | <0.001 |
| **No. of live births** | 356 | 1 (1-4) | 2 (1-8) | 2 (1-12) | 2 (1-4) | <0.001 |
| **No. of miscarriages** | 356 | 0 (0-4) | 0 (0-3) | 0 (0-2) | 1 (0-3) | 0.049 |
| **Family anamnesis of CHD** | 358 | | | | | |
| Negative | | 194 (97.5%) | 75 (81.5%) | 29 (74.4%) | 22 (87.6%) | $6.4 \times 10^{-8}$ |
| Positive | | 5 (2.5%) | 17 (18.5%) | 10 (25.6%) | 6 (21.4%) | |

| | | | | | | |
|---|---|---|---|---|---|---|
| **Maternal diabetes at conception** | 358 | | | | | |
| No | | 199 (100%) | 92 (100%) | 39 (100%) | 28 (100%) | -- |
| Yes | | 0 (0%) | 0 (0%) | 0 (0%) | 0 (0%) | |
| **Gestational diabetes** | 358 | | | | | |
| No | | 171 (85.9%) | 78 (84.8%) | 35 (89.7%) | 26 (92.9%) | 0.737 |
| Yes | | 28 (14.1%) | 14 (15.2%) | 4 (10.3%) | 2 (7.1%) | |
| **Maternal chronic disease** | 358 | | | | | |
| No | | 184 (92.5%) | 72 (78.3%) | 31 (79.5%) | 26 (92.9%) | 0.002 |
| Yes | | 15 (7.5%) | 20 (21.7%) | 8 (20.5%) | 2 (7.1%) | |
| **Antiepileptics in pregnancy** | 358 | | | | | |
| No | | 198 (99.5%) | 92 (100%) | 39 (100%) | 28 (100%) | 1.000 |
| Yes | | 1 (0.5%) | 0 (0%) | 0 (0%) | 0 (0%) | |
| **Other drugs in pregnancy** | 358 | | | | | |
| No | | 107 (53.8%) | 32 (34.8%) | 26 (66.7%) | 15 (53.6%) | 0.003 |
| Yes | | 92 (46.2%) | 60 (65.2%) | 13 (33.3%) | 13 (46.4%) | |
| **Fever in pregnancy** | 358 | | | | | |
| No | | 186 (93.5%) | 88 (95.7%) | 37 (94.9%) | 27 (96.4%) | 0.943 |
| Yes | | 13 (6.5%) | 4 (4.3%) | 2 (5.1%) | 1 (3.6%) | |
| **Sauna in pregnancy** | 358 | | | | | |
| No | | 194 (97.5%) | 91 (98.9%) | 39 (100%) | 28 (100%) | 0.905 |
| Yes | | 5 (2.5%) | 1 (1.1%) | 0 (0.0%) | 0 (0.0%) | |
| **Folate supplement initiation** | 353 | | | | | |
| No folate supplement | | 19 (9.8%) | 24 (26.1%) | 9 (23.1%) | 6 (21.4%) | 0.001 |
| Before 3 weeks post-conception | | 113 (58.2%) | 44 (47.8%) | 12 (30.8%) | 15 (53.6%) | |
| After 3 weeks post-conception | | 62 (32%) | 24 (26.1%) | 18 (46.2%) | 7 (25.0%) | |
| ***Folate supplementation in pregnancy: folate formulation*** | 257 | | | | | |
| Folic acid | | 134 (88.2%) | 57 (91.9%) | 22 (91.7%) | 17 (89.5%) | 0.879 |
| Metafolin | | 18 (11.8%) | 5 (8.1%) | 2 (8.3%) | 2 (10.5%) | (α = 0.05) |
| **Other supplements in pregnancy[d]** | 358 | | | | | |
| No | | 158 (79.4%) | 65 (70.7%) | 31 (79.5%) | 20 (71.4%) | 0.339 |
| Yes | | 41 (20.6%) | 27 (29.3%) | 8 (20.5%) | 8 (28.6%) | |

| | | | | | | |
|---|---|---|---|---|---|---|
| **Methionine per month intake (g)** | 282 | 47 (18-847) | 34 (8-84) | 44 (5-488) | 34 (7-68) | <0.001 |
| **Folic acid per month intake (mg)** | 282 | 23 (8-511) | 19 (4-84) | 18 (5-333) | 20 (4-36) | <0.001 |
| **Maternal genotype SLC19A1** | 353 | | | | | |
| GG | | 70 (35.2%) | 32 (35.6%) | 11 (28.9%) | 6 (23.1%) | 0.599 |
| AG or AA | | 129 (64.8%) | 58 (64.4%) | 27 (71.1%) | 20 (76.9%) | |
| **Child genotype SLC19A1** | 354 | | | | | |
| GG | | 54 (27.1%) | 33 (36.7%) | 16 (41.0%) | 4 (15.4%) | 0.056 |
| AG or AA | | 145 (72.9%) | 57 (63.3%) | 23 (59.0%) | 22 (84.6%) | |
| **Maternal genotype DHFR** | 354 | | | | | |
| CC | | 110 (55.3%) | 52 (57.1%) | 23 (59.0%) | 15 (60.0%) | 0.954 |
| CA or AA | | 89 (44.7%) | 39 (42.9%) | 16 (41.0%) | 10 (40.0%) | |
| **Child genotype DHFR** | 355 | | | | | |
| CC | | 111 (55.8%) | 46 (50.5%) | 25 (64.1%) | 16 (61.5%) | 0.489 |
| CA or AA | | 88 (44.2%) | 45 (49.5%) | 14 (35.9%) | 10 (38.5%) | |
| **Maternal genotype FPGS** | 355 | | | | | |
| CC or CT | | 156 (78.4%) | 73 (79.3%) | 27 (78.4%) | 24 (92.3%) | 0.222 |
| TT | | 43 (21.6%) | 19 (20.7%) | 11 (21.6%) | 2 (7.7%) | |
| **Child genotype FPGS** | 354 | | | | | |
| CC or CT | | 169 (84.9%) | 72 (79.1%) | 35 (92.1%) | 21 (80.8%) | 0.283 |
| TT | | 30 (15.1%) | 19 (20.9%) | 3 (7.9%) | 5 (19.2%) | |
| **Maternal genotype BHMT** | 354 | | | | | |
| GG or AG | | 184 (92.5%) | 82 (89.1%) | 36 (94.7%) | 22 (88.0%) | 0.608 |
| AA | | 15 (7.5%) | 10 (10.9%) | 2 (5.3%) | 3 (12.0%) | |
| **Child genotype BHMT** | 352 | | | | | |
| GG or AG | | 182 (91.9%) | 82 (90.1%) | 31 (83.8%) | 25 (96.2%) | 0.358 |
| AA | | 16 (8.1%) | 9 (9.9%) | 6 (16.2%) | 1 (3.8%) | |
| **Maternal genotype GNMT** | 355 | | | | | |
| CC or CT | | 161(80.9%) | 62 (67.4%) | 31 (79.5%) | 16 (64.0%) | 0.034 |
| TT | | 38 (19.1%) | 30 (32.6%) | 8 (20.5%) | 9 (36.0%) | |
| **Child genotype GNMT** | 352 | | | | | |
| CC | | 65 (32.7%) | 23 (25.6%) | 12 (31.6%) | 4 (16.0%) | 0.283 |
| CT or TT | | 134 (67.3%) | 67 (74.4%) | 26 (68.4%) | 21 (84.0%) | |
| **Maternal genotype DNMT3B** | 354 | | | | | |

| | | | | | | |
|---|---|---|---|---|---|---|
| CC | | 57 (28.6%) | 39 (42.4%) | 7 (18.4%) | 6 (24.0%) | 0.026 |
| CT or TT | | 142 (71.4%) | 53 (57.6%) | 31 (81.6%) | 19 (76.0%) | |
| **Child genotype DNMT3B** | 354 | | | | | |
| CC | | 58 (29.1%) | 33 (35.9%) | 9 (24.3%) | 9 (34.6%) | 0.517 |
| CT or TT | | 141 (70.9%) | 59 (64.1%) | 28 (75.7%) | 17 (65.4%) | |
| **Maternal genotype MTHFD1** | 353 | | | | | |
| GG | | 68 (34.3%) | 30 (33.7%) | 10 (25.6%) | 12 (44.4%) | 0.475 |
| AG or AA | | 130 (65.7%) | 59 (66.3%) | 29 (74.4%) | 15 (55.6%) | |
| **Child genotype MTHFD1** | 354 | | | | | |
| GG | | 54 (27.3%) | 31 (34.4%) | 10 (26.3%) | 16 (57.1%) | 0.015 |
| AG or AA | | 144 (72.7%) | 59 (65.6%) | 28 (73.7%) | 12 (42.9%) | |
| **Maternal genotype MTHFR** | 355 | | | | | |
| 677 CC/1298 AA | | 30 (15.2%) | 12 (13.2%) | 2 (5.1%) | 5 (18.5%) | 0.321 |
| At least one mutated allele | | 168 (84.8%) | 79 (86.8%) | 37 (94.9%) | 22 (81.5%) | |
| **Child genotype MTHFR** | 356 | | | | | |
| 677 CC/1298 AA | | 16 (8.1%) | 11 (12.0%) | 4 (10.5%) | 6 (21.4%) | 0.161 |
| At least one mutated allele | | 182 (91.9%) | 81 (88.0%) | 34 (89.5%) | 22 (78.6%) | |
| **Maternal genotype MTRR** | 356 | | | | | |
| AA or AG | | 129 (65.2%) | 60 (65.2%) | 22 (56.4%) | 22 (81.5%) | 0.206 |
| GG | | 69 (34.8%) | 32 (34.8%) | 17 (43.6%) | 5 (18.5%) | |
| **Child genotype MTRR** | 355 | | | | | |
| AA | | 30 (15.2%) | 11 (12.1%) | 6 (15.8%) | 9 (32.1%) | 0.109 |
| AG or GG | | 168 (84.8%) | 80 (87.9%) | 32 (84.2%) | 19 (67.9%) | |
| **No. of mutated alleles in mother-child pairs**[e] | 333 | 16 (8-27) | 17 (7-27) | 16 (11-25) | 15 (9-19) | 0.166 |

Italic text, variables tested only in subgroups; underlined, variables included in multinomial logistic regression models. Discrepancies between the total numbers of subjects for different variables are due to the missing questionnaire data or unsuccessful genotyping.

[a], Gaussian parametric variables: for simple statistical analysis (unadjusted p) one-way ANOVA was used.

[b], Non-Gaussian parametric and rank/score variables: for simple statistical analysis (unadjusted p) the Kruskal-Wallis test was used.

[c], Categorical variables: for simple statistical analysis (unadjusted p) the Fisher`s exact test was used.

[d], Multivitamin, Minerals (Mg, Ca), Omega 3.

[e], Minimum: 0, Maximum: 40.

--, not applicable. Statistics could not be calculated.
